# Preditores de Mortalidade Hospitalar nos Pacientes Tratados por Angioplastia Primária: Um Estudo de Caso-Controle Multicêntrico

**DOI:** 10.36660/abc.20210015

**Published:** 2022-07-28

**Authors:** Pedro Paulo Neves de Castro, Marco Antonio Nazaré Castro, Guilherme Abreu Nascimento, Isabel Moura, José Luiz Barros Pena

**Affiliations:** 1 Hospital Marcio Cunha Ipatinga MG Brasil Hospital Marcio Cunha – Hemodinâmica, Ipatinga, MG – Brasil; 2 Faculdade de Ciências Médicas de Minas Gerais Programa de Pós-Graduação stricto sensu em Ciências da Saúde Belo Horizonte MG Brasil Faculdade de Ciências Médicas de Minas Gerais – Programa de Pós-Graduação stricto sensu em Ciências da Saúde, Belo Horizonte, MG – Brasil; 3 Unimed Vale do Aço Ipatinga MG Brasil Unimed Vale do Aço, Ipatinga, MG – Brasil; 4 Hospital Felício Rocho Belo Horizonte MG Brasil Hospital Felício Rocho – Ecocardiografia, Belo Horizonte, MG – Brasil

**Keywords:** Infarto Agudo do Miocárdio, Banco de Dados, Reperfusão Miocárdica, Angioplastia Primária, Mortalidade

## Abstract

**Fundamento:**

A estratificação do risco de morte dos pacientes no contexto da angioplastia primária (ATC) é fundamental.

**Objetivo:**

Identificar os fatores relacionados ao desfecho morte em pacientes submetidos a ATC.

**Métodos:**

Estudo de caso-controle, utilizando como fonte de dados um registro brasileiro. A associação entre cada variável e o desfecho óbito foi avaliada via modelo de regressão logística binária. Consideramos significativo p<0,05.

**Resultados:**

Foram analisados 26.990 registros, sendo 18.834 (69,8%) do sexo masculino, com idade mediana de 61 (17) anos. Na análise multivariada, as principais variáveis relacionadas ao desfecho óbito com seus respectivos *odds ratio* e intervalos de confiança (IC) com nível de significância de 95% foram a idade avançada 70 - 79 anos (2,46; 1,64 - 3,79) e ≥ 80 anos (3,68; 2,38 - 5,81), p<0,001, classificação de Killip II (2,71; 1,92 - 3,83), Killip III (8,14; 5,67 - 11,64), Killip IV (19,83; 14,85 - 26,69), p<0,001, disfunção global acentuada do ventrículo esquerdo (VE) (3,63; 2,39 - 5,68), p<0,001 e ocorrência de infarto após a intervenção (5,01; 2,57- 9,46), p<0,001. O principal fator protetor foi o fluxo TIMI III pós-intervenção (0,18; 0,13 - 0,24), p<0,001, seguido do TIMI II (0,59; 0,41 - 0,86), p=0,005, sexo masculino (0,79; 0,64 - 0,98), p= 0,032, dislipidemia (0,69; 0,59 - 0,85), p<0,001 e número de lesões tratadas (0,86; 0,9 - 0,94), p<0,001.

**Conclusão:**

Os preditores de mortalidade nos pacientes submetidos a ATC foram: classificação de Killip, reinfarto, idade, disfunção global acentuada do VE, sexo feminino e fluxo TIMI 0/I pós-intervenção.

## Introdução

As doenças cardiovasculares (DCV) são a principal causa de morbimortalidade no Brasil. Em termos relativos, as condições cardíacas representaram 8,3% de todas as internações e 18,6% de todo o reembolso de despesas hospitalares do sistema único de saúde (SUS). A doença isquêmica do coração é a que causa o maior número de mortes dentre as DCV.^1^

O acesso ao tratamento visando ao restabelecimento do fluxo coronariano é fundamental na redução da mortalidade por infarto agudo do miocárdio com supradesnivelamento do segmento ST (IAMCSST). Estudos com uso de aspirina, associada a medicações fibrinolíticas, mostraram significativa redução na mortalidade precoce.^2 - 4^

Outro método de tratamento, a angioplastia primária (ATC) consiste na desobstrução mecânica da artéria relacionada ao IAMCSST, sendo esta a estratégia de tratamento preferencial, desde que possa ser realizada em tempo hábil, ou seja, até noventa minutos, por equipe experiente.^5 - 7^ A ATC, quando comparada a fibrinólise química, é considerada o tratamento mais efetivo, podendo reduzir as taxas de mortalidade, recorrência do infarto não fatal e acidente vascular cerebral.^8^

A identificação dos pacientes de maior risco é fundamental para informações prognósticas, auxiliando no processo de decisão médica. O conhecimento dessas variáveis, marcadoras de pior prognóstico, pode ajudar na seleção de grupos de pacientes com maior taxa de eventos para futuros estudos, ajustar as características basais da população em estudos epidemiológicos e gerar hipóteses para outros estudos.^9 , 10^

Existem várias publicações com modelos para estratificação de risco, porém são poucos os dados na população brasileira.^11 - 16^ Em 1991, foi criada a Central Nacional de Intervenções Cardiovasculares (CENIC), um banco de dados oficial da Sociedade Brasileira de Hemodinâmica e Cardiologia Intervencionista (SBHCI). Esse banco de dados traz informações que são provenientes da contribuição espontânea dos seus associados. O registro já foi caracterizado previamente e utilizado em outras publicações.^17 - 19^

O objetivo do presente estudo é identificar os fatores de risco para morte nos pacientes submetidos a ATC.

## Métodos

Estudo de caso-controle, multicêntrico. Utilizamos uma fonte de dados secundária (CENIC). Os pacientes foram divididos em dois grupos, no primeiro (casos) foram alocados os dados dos pacientes que evoluíram a óbito (por qualquer causa) e no segundo grupo (controle), os pacientes submetidos ao procedimento e que sobreviveram. Os dados foram coletados durante o período de hospitalização.

### População

Foram selecionados registros de pacientes submetidos a ATC primária, no período de janeiro de 2004 a dezembro de 2018. Foram excluídas as informações de pacientes com idade inferior a 18 anos ou não informada, com falta de dados sobre óbito hospitalar e relato de uso prévio de trombolíticos ou ausência dessa informação.

Excluímos também registros de procedimentos que não são aprovados para angioplastia primária de acordo com a Diretriz da Sociedade Brasileira de Cardiologia,^7^ sendo excluídos casos que utilizaram dispositivos de aterectomia rotacional, direcional, *cutting balloon* e *excimer lazer* . Ao todo, 109 registros relataram pelo menos uma dessas técnicas.

**Figura 1:**
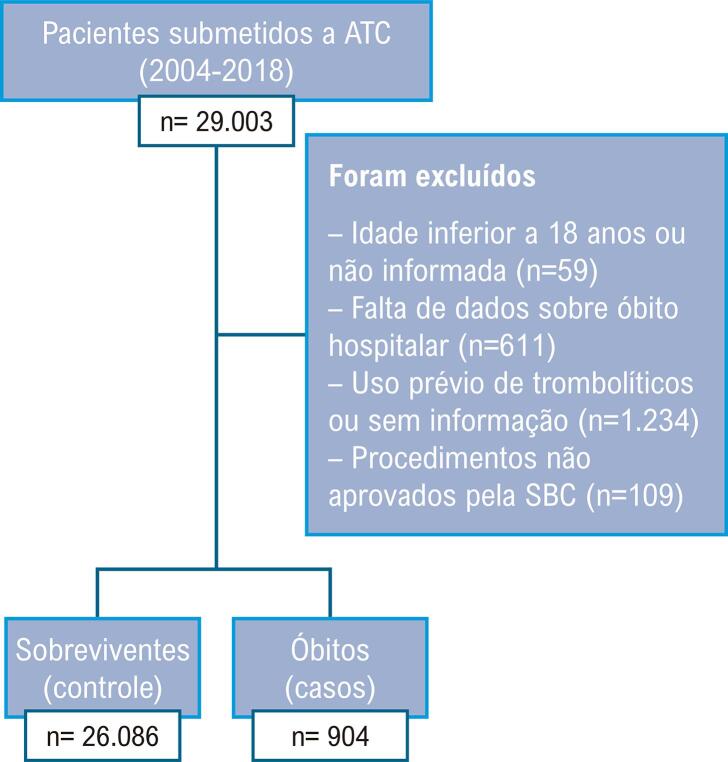
População, critérios de exclusão e distribuição de casos e controles. ATC: angioplastia primária; SBC: Sociedade Brasileira de Cardiologia.

De um total de 29.003 registros originais, foram incluídos na análise 26.990. O fluxograma com a população do estudo, os critérios de exclusão e a distribuição de casos e controles é apresentado na Figura 1 .

## Definições

Foram incluídos pacientes com critérios clínicos e eletrocardiográficos compatíveis com o diagnóstico de IAMCSST, selecionados para uma estratégia de angioplastia primária. O diagnóstico foi confirmado pela angiografia em todos os casos. A decisão de inclusão dos pacientes no registro foi a critério do cardiologista intervencionista.

A análise referente às variáveis angiográficas, incluindo a função ventricular foi realizada pela estimativa visual dos operadores, e as definições utilizadas seguiram a Diretriz de Intervenção Coronária Percutânea e Métodos Adjuntos Diagnósticos em Cardiologia Intervencionista da SBHCI.^20^

A escolha do acesso vascular, uso de medicações adjuvantes e das técnicas do procedimento foi de livre escolha dos operadores.

O registro CENIC é gerido pela empresa Coreware (www.coreware.com.br), que realizou a extração dos dados para a pesquisa, sem identificação dos participantes e respectivos hospitais de origem, visando à preservação do sigilo.

As variáveis foram selecionadas baseando-se em publicações prévias.^10 - 16^

### Análise estatística

As variáveis qualitativas foram apresentadas como frequências, e as quantitativas, como mediana (distância interquartílica). As variáveis quantitativas foram submetidas ao teste de normalidade de Kolmogorov-Smirnov. A comparação das taxas de mortalidade entre sexos foi avaliada via teste Qui-Quadrado. A associação entre cada uma das variáveis preditoras e o desfecho óbito foi avaliada via modelo de regressão logística simples. A análise univariada foi feita com todas as variáveis apresentadas na Tabela 1 . Essas variáveis foram selecionadas com base em estudos prévios. As variáveis com p<0,20 na análise univariada foram incluídas em um modelo de regressão logística binária multivariado. A partir da estratégia *stepwise* chegou-se ao modelo final, e a qualidade do ajuste foi avaliada utilizando-se o teste de Hosmer-Lemeshow. Todos os dados faltantes foram excluídos da análise estatística.

**Tabela 1:** Características da amostra e associação com o desfecho óbito

Características	Toda a amostra (n=26.990)	Não (n=26.086)	Sim (n=904)	p-valor	OR (IC 95%)
**Gênero**					
F	8.156 (30,2%)	7.764 (29,8%)	392 (43,4%)	-	-
M	18.834 (69,8%)	18.322 (70,2%)	512 (56,6%)	<0,001	0,55 (0,48; 0,63)
**Idade**					
19 a 49 anos	4.472 (16,6%)	4.400 (16,9%)	72 (8,0%)	-	-
50 a 59 anos	7.886 (29,2%)	7.734 (29,6%)	152 (16,8%)	0,204	1,20 (0,91; 1,60)
60 a 69 anos	7.395 (27,4%)	7.166 (27,5%)	229 (25,3%)	<0,001	1,95 (1,50; 2,57)
70 a 79 anos	4.968 (18,4%)	4.714 (18,1%)	254 (28,1%)	<0,001	3,29 (2,54; 4,32)
≥ 80 anos	2.269 (8,4%)	2.072 (7,9%)	197 (21,8%)	<0,001	5,81 (4,44; 7,69)
Killip* (n=26.989)					
I	20.560 (76,2%)	20.359 (78,0%)	201 (22,2%)	-	-
II	3.560 (13,2%)	3.452 (13,2%)	108 (11,9%)	<0,001	3,17 (2,49; 4,01)
III	1.079 (4,0%)	969 (3,7%)	110 (12,2%)	<0,001	11,50 (9,01; 14,60)
IV	1.790 (6,6%)	1.305 (5,0%)	485 (53,7%)	<0,001	37,64 (31,69; 44,86)
Localização das lesões* (n=27.179)					
DA proximal	7.266 (26,9%)	6.951 (26,6%)	315 (34,8%)	-	-
Coronária direita médio/distal e ramos	6.451 (23,9%)	6.326 (24,3%)	125 (13,8%)	<0,001	0,44 (0,35; 0,54)
DA médio/distal e ramos	5.515 (20,4%)	5.379 (20,6%)	136 (15,0%)	<0,001	0,56 (0,45; 0,68)
Coronária direita proximal	3.696 (13,7%)	3.561 (13,7%)	135 (14,9%)	0,089	0,84 (0,68; 1,03)
Circunflexa distal/ramos	1.989 (7,4%)	1.949 (7,5%)	40 (4,4%)	<0,001	0,45 (0,32; 0,62)
Circunflexa proximal	1.486 (5,5%)	1.423 (5,5%)	63 (7,0%)	0,869	0,98 (0,73; 1,28)
Enxertos	370 (1,4%)	345 (1,3%)	25 (2,8%)	0,029	1,60 (1,02; 2,39)
Tronco	217 (0,8%)	152 (0,6%)	65 (7,2%)	<0,001	9,44 (6,87; 12,83)
Extensão da doença* (n=26.751)					
Uniarterial	12.699 (47,5%)	12.484 (48,3%)	215 (24,0%)	-	-
Biarterial	7.889 (29,5%)	7.610 (29,4%)	279 (31,1%)	<0,001	2,13 (1,78; 2,55)
Multiarterial + TCE	36 (0,1%)	29 (0,1%)	7 (0,8%)	<0,001	14,02 (5,60; 30,59)
TCE	44 (0,2%)	29 (0,1%)	15 (1,7%)	<0,001	30,03 (15,48; 55,99)
Triarterial	6.083 (22,7%)	5.702 (22,1%)	381 (42,5%)	<0,001	3,88 (3,28; 4,61)
Tempo porta-balão^1^* (minutos) (n=25.837)	70,00 (75,00)	70,00 (75,00)	80,00 (66,80)	0,010	1,0006 (1,0001; 1,001)
Cirurgia prévia CRVM	803 (3,0%)	759 (2,9%)	44 (4,9%)	<0,001	1,71 (1,23; 2,30)
Angioplastia prévia	3.143 (11,6%)	3.044 (11,7%)	99 (11,0%)	0,508	0,93 (0,75; 1,14)
IAM prévio* (n=26.957)	2.948 (10,9%)	2.808 (10,8%)	140 (15,5%)	<0,001	1,52 (1,26; 1,82)
Diabetes Mellitus	5.270 (19,5%)	5.021 (19,2%)	249 (27,5%)	<0,001	1,59 (1,37; 1,85)
Insulino-dependentes	753 (2,8%)	697 (2,7%)	56 (6,2%)	<0,001	2,41 (1,80; 3,16)
HAS	19.045 (70,6%)	18.406 (70,6%)	639 (70,7%)	0,934	1,006 (0,87; 1,17)
IRA	43 (0,2%)	25 (0,1%)	18 (2,0%)	<0,001	21,18 (11,35; 38,75)
Tabagismo	9.521 (35,3%)	9.273 (35,5%)	248 (27,4%)	<0,001	0,69 (0,59; 0,79)
Dislipidemia	13.221 (49,0%)	12.825 (49,2%)	396 (43,8%)	0,002	0,81 (0,70; 0,92)
História familiar	6.364 (23,6%)	6.208 (23,8%)	156 (17,3%)	<0,001	0,67 (0,56; 0,79)
**TIMI Pré**					
0	18.160 (67,3%)	17.472 (67,0%)	688 (76,1%)	-	-
1	1.576 (5,8%)	1.513 (5,8%)	63 (7,0%)	0,678	1,06 (0,81; 1,36)
2	2.435 (9,0%)	2.371 (9,1%)	64 (7,1%)	0,004	0,69 (0,52; 0,88)
3	4.819 (17,9%)	4.730 (18,1%)	89 (9,8%)	<0,001	0,48 (0,38; 0,59)
TIMI Pós* (n=26.975)					
0	1.175 (4,4%)	955 (3,7%)	220 (24,4%)	-	-
1	322 (1,2%)	257 (1,0%)	65 (7,2%)	0,554	1,10 (0,80; 1,49)
2	1.289 (4,8%)	1.146 (4,4%)	143 (15,9%)	<0,001	0,54 (0,43; 0,68)
3	24.189 (89,7%)	23.715 (91,0%)	474 (52,5%)	<0,001	0,09 (0,07; 0,10)
Diâmetro do vaso^1^* (n=19.931)	3,00 (0,75)	3,00 (0,75)	3,00 (0,75)	<0,001	0,63 (0,52; 0,76)
Função VE* (n=16.880)					
Normal	3.169 (18,8%)	3.139 (19,2%)	30 (6,0%)	-	-
Disfunção global leve	6.167 (36,5%)	6.123 (37,4%)	44 (8,8%)	0,230	0,75 (0,47; 1,21)
Disfunção global moderada	5.230 (31,0%)	5.130 (31,3%)	100 (20,0%)	<0,001	2,04 (1,37; 3,13)
Disfunção global acentuada	2.314 (13,7%)	1.989 (12,1%)	325 (65,1%)	<0,001	17,10 (11,92; 25,47)
Complicação vascular menor	87 (0,3%)	82 (0,3%)	5 (0,6%)	0,219	1,76 (0,62; 3,94)
Complicação vascular maior	31 (0,1%)	25 (0,1%)	6 (0,7%)	<0,001	6,97 (2,58; 15,94)
AVC hemorrágico	16 (0,1%)	12 (<0,1%)	4 (0,4%)	<0,001	9,66 (2,70; 27,78)
AVC isquêmico	17 (0,1%)	11 (<0,1%)	6 (0,7%)	<0,001	15,84 (5,45; 41,72)
Via de acesso* (n=25.032)					
Femoral	19.278 (77,0%)	18.690 (76,7%)	588 (86,6%)	-	
Braquial – dissecção	299 (1,2%)	291 (1,2%)	8 (1,2%)	0,709	0,87 (0,39; 1,66)
Braquial – punção	165 (0,7%)	162 (0,7%)	3 (0,4%)	0,364	0,59 (0,15; 1,55)
Radial	5.290 (21,1%)	5.210 (21,4%)	80 (11,8%)	<0,001	0,49 (0,38; 0,61)
Abciximab* (n=25.107)	830 (3,3%)	800 (3,3%)	30 (4,4%)	0,103	1,36 (0,92; 1,94)
Tirofiban* (n=25.107)	3.199 (12,7%)	3.067 (12,6%)	132 (19,4%)	<0,001	1,68 (1,38; 2,03)
AAS* (n=25.107)	22.475 (89,5%)	21.873 (89,5%)	602 (88,5%)	0,394	0,90 (0,71; 1,15)
Calcificação	5.448 (20,2%)	5.176 (19,8%)	272 (30,1%)	<0,001	1,74 (1,50; 2,01)
Trombo intracoronariano	16.812 (62,3%)	16.197 (62,1%)	615 (68,0%)	<0,001	1,30 (1,13; 1,50)
Infarto após intervenção	130 (0,5%)	98 (0,4%)	32 (3,5%)	<0,001	9,73 (6,40; 14,42)
Lesões tratadas^1^	1,00 (1,00)	1,00 (1,00)	1,00 (0,00)	<0,001	0,84 (0,79; 0,89)

*variáveis que apresentaram missings, n válido entre parênteses. ^1^Dados apresentados como mediana (distância interquartílica). Os p-valores referem-se ao modelo logístico binário simples. Da: artéria descendente anterior; TCE: tronco da coronária esquerda; CRVM: cirurgia de revascularização do miocárdio; HAS: hipertensão arterial sistêmica; IRA: insuficiência renal aguda; VE: ventrículo esquerdo; AVC: acidente vascular cerebral; AAS: ácido acetilsalicílico. Fonte: elaboração do autor, 2021.

Os resultados foram apresentados como *odds ratio* (OR) com respectivos intervalos de 95% de confiança (IC 95%). As análises foram realizadas no programa gratuito R versão 4.0.0, e foi considerado significativo p<0,05.

### Aspectos éticos

O trabalho foi aprovado pelo Comitê de Ética da Faculdade Ciências Médicas de Minas Gerais, protocolo número 3.502.883. Houve dispensa do termo de consentimento livre e esclarecido (TCLE). Todos os procedimentos realizados neste estudo estão em conformidade com o descrito na resolução 466/2012.

## Resultados

**Figura 2:**
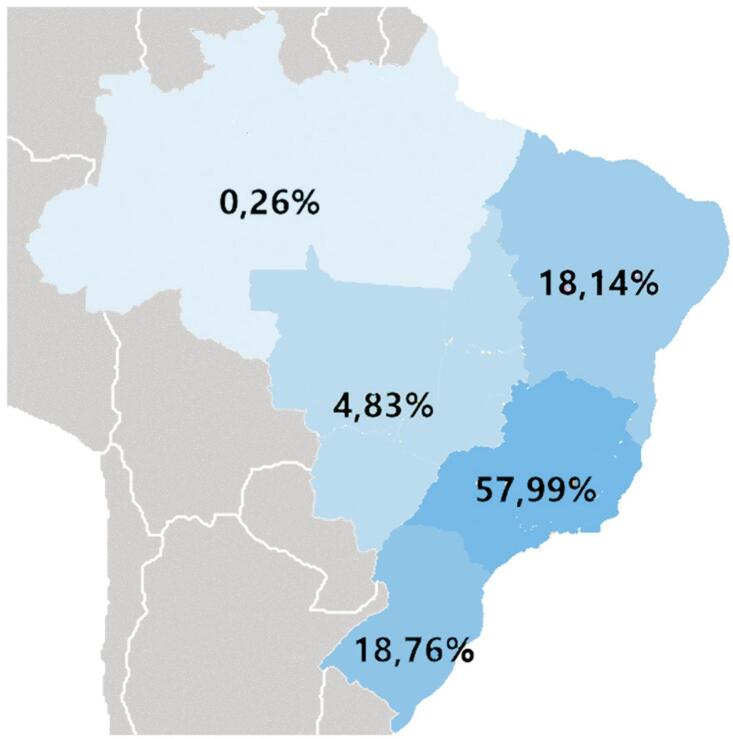
Distribuição de casos por região.

Foram analisados 26990 registros provenientes de todas as regiões brasileiras, a distribuição de casos é apresentada na Figura 2 . A maioria dos registros, 1883 (69,8%) foram do sexo masculino, a idade mediana encontrada foi 61 (DI 17) anos e o fator de risco mais frequente foi a hipertensão arterial sistêmica, sendo relatada por 19045 (70,6%) dos participantes.

A maioria dos pacientes, 20560 (76,2%) apresentava classificação de Killip classe I com predomínio de doença acometendo um único vaso, 12699 (47,5%) e apresentando disfunção ventricular leve, 6167 (36,5%).

O número total de óbitos foi de 904 pacientes e a taxa de mortalidade geral foi 3,3%. A taxa de mortalidade foi menor nos homens em relação às mulheres (2,7% e 4,8% respectivamente, p<0,001, teste Qui-Quadrado).

A Tabela 1 mostra as características da amostra, a associação com o desfecho óbito e os resultados do teste de associação e as estimativas do *odds ratio* (OR) com intervalo de confiança (IC) de 95%, com seus respectivos valores p, obtidos por meio do ajuste de modelos logísticos univariados. Nesta análise, as variáveis com p <0,20 utilizadas na modelo múltiplo foram gênero, idade, classificação de Killip, localização da lesões, extensão da doença coronariana, tempo porta-balão, histórico de cirurgia de revascularização do miocárdio, relato de infarto prévio, diabetes, tabagismo, dislipidemia, hipertensão arterial sistêmica, histórico familiar de doença coronariana precoce, classificação do fluxo TIMI antes e após a intervenção, diâmetro do vaso, desenvolvimento de complicações vasculares maiores, insuficiência renal, acidente vascular encefálico isquêmico ou hemorrágico, novo infarto pós intervenção, acesso vascular, número de lesões tratadas e presença de calcificação e trombo.

A Tabela 2 apresenta os fatores relacionados ao desfecho óbito, com respectivos resultados das estimativas da OR, intervalos de 95% de confiança e valores p, obtidos por meio do ajuste pelo modelo de regressão logística binária multivariado.

**Tabela 2:** Variáveis que se correlacionaram de forma significativa e independente com óbito intra-hospitalar

Característica	OR	IC 95% OR	p-valor
Intercepto	0,021	(0,011; 0,039)	<0,001
Gênero M	0,789	(0,635; 0,981)	0,032
**Idade (ref. <50)**			
50 a 59	1,625	(1,059; 2,540)	0,029
60 a 69	2,004	(1,336; 3,076)	0,001
70 a 79	2,462	(1,635; 3,789)	<0,001
≥ 80	3,688	(2,384; 5,812)	<0,001
**Killip (ref. I)**			
II	2,718	(1,919; 3,827)	<0,001
III	8,139	(5,672; 11,637)	<0,001
IV	19,833	(14,851; 26,688)	<0,001
Dislipidemia	0,689	(0,558; 0,850)	<0,001
**TIMI Pós (ref. 0)**			
1	1,303	(0,774; 2,162)	0,313
2	0,593	(0,409; 0,857)	0,005
3	0,176	(0,133; 0,235)	<0,001
**Função VE (ref. Normal)**			
Disfunção global leve	0,799	(0,491; 1,322)	0,373
Disfunção global moderada	1,206	(0,782; 1,914)	0,410
Disfunção global acentuada	3,625	(2,393; 5,675)	<0,001
Infarto após intervenção	5,006	(2,568; 9,460)	<0,001
N° de lesões tratadas	0,859	(0,785; 0,938)	<0,001

*P-valor Hosmer-Lemeshow 0,683. VE: ventrículo esquerdo. Fonte: elaboração do autor, 2021.*

## Discussão

Os principais indicadores de mortalidade nos pacientes submetidos a ATC primária encontrados no nosso estudo, além da idade e sexo feminino, foram relacionados ao impacto do infarto na função ventricular, como a classificação de Killip e a presença de disfunção global acentuada do VE analisada pela angiografia. Por outro lado, a presença de fluxo TIMI II/III após a intervenção, refletiu o sucesso do tratamento, que visa justamente à manutenção da função ventricular e a prevenção de outras complicações cardiovasculares. A ocorrência de reinfarto foi infrequente, mas mostrou-se como um indicador independente de mortalidade nesses pacientes.

As taxas de mortalidade nos pacientes submetidos a ATC variam de 2,3% a 11,9% de acordo com diferentes fontes.^15 , 21 - 24^ No registro CENIC, encontramos uma taxa de óbitos de 3,4%. Esse achado pode ser relativo à subnotificação e ao menor risco da amostra. Na Tabela 3 , demonstramos comparação entre as variáveis correlacionadas ao desfecho morte, estabelecidas no nosso estudo, em relação a outros já publicados na literatura.^11 - 14 , 25 , 26^

**Tabela 3:** Comparativo de variáveis relacionadas ao desfecho óbito

	CENIC (n=26.990)	DynTIMI (n=20.506)	PAMI (n=3.252)	CADILLAC (n=2.082)	GRACE (n=11.389)	Zwolle (n=1.791)	ALPHA (n=1.255)
**Tempo**	**Hospitalar**	**1 ano**	**6 meses**	**1 ano**	**6 meses**	**30 dias**	**30 dias**
Idade	+	+	+	+	+	+	+
Gênero feminino	+						
Hipotensão Arterial		+			+		+
Frequência Cardíaca		+	+		+		+
Classificação de Killip	+	+	+	+	+		
Diabetes mellitus			+				
HAS							
Angina pectoris							
IAM Anterior ou BRE		+	+			+	
Peso		+				+	
Tempo de isquemia		+					
Fluxo (TIMI final de 0 a 2)	+			+		+	
FEVE				+			
Disfunção acentuada VE	+						
Anemia				+			
Doença nos três vasos				+		+	
Desvio do segmento ST					+		
Creatinina/IR		+		+	+		
Parada cardíaca					+		+
Marcadores de lesão miocárdica					+		
Recorrência de infarto	+	+					
AVC		+					
Arritmia		+					
IC/Choque		+				+	
Sangramento maior		+					
Acesso femoral							+

*CENIC: Central Nacional de Intervenções Cardiovasculares; dynTIMI: dynamic Thrombolysis In Myocardial Infarction; PAMI: Primary Angioplasty in Myocardial Infarction; CADILLAC: Controlled Abciximab and Device Investigation to Lower Late Angioplasty Complications; GRACE: Global Registry of Acute Coronary Events; ALPHA: (Age, Life support, Pressure, Heart rate, Access site); HAS: hipertensão arterial sistêmica; IAM: infarto agudo do miocárdio; BRE, bloqueio do Ramo Esquerdo; FEVE, fração de ejeção do ventrículo esquerdo; VE: ventrículo esquerdo; PAS: pressão arterial sistólica; IR: insuficiência renal; AVC: acidente vascular cerebral; IC: insuficiência cardíaca. Fonte: elaboração do autor, 2021.*

Observa-se que o único indicador do estudo CENIC divergente dos demais modelos de risco apresentados na Tabela 3 foi o sexo feminino, porém, esse achado já foi relatado por outras publicações.^27 , 28^

Alguns autores relacionam a maior presença de sintomas atípicos nas mulheres com atrasos no tratamento, a chamada síndrome de Yentl. A angioplastia também pode ser mais desafiadora, levando a uma menor taxa de sucesso.^29^ Dados ausentes no nosso banco de dados, como o tempo total de isquemia, a ocorrência de sangramento fora do sítio de acesso e peso poderiam explicar em parte a pior evolução no sexo feminino.

A classificação de Killip e Kimball, foi a variável que se mostrou como melhor indicador de prognóstico, fato corroborado por outros estudos.^10 , 12 , 13^ No registro Grace, a chance óbito aumentava cerca de 3 vezes a cada incremento na classificação de Killip 3,30 (3,00-3,60) p <0.001. Na nossa casuística tivemos 1790 casos (6,6% do total) com choque cardiogênico (classe IV de Killip), semelhante à incidência descrita na literatura (5 a 10%).^30^

A falência ventricular é a principal causa de morte nesses pacientes e o único tratamento que tem mostrado efetividade é a reperfusão precoce. O uso de dispositivos de assistência ventricular, como o balão intra-aórtico, apresenta resultados conflitantes.^31^ Outros dispositivos têm sido testados e até utilizados na prática clínica, porém ainda sem estudos conclusivos publicados.^32^

O objetivo da intervenção é a obtenção do fluxo final TIMI III. Esse resultado relacionou-se fortemente com a redução nas chances de óbito OR 0,18 (IC 0,13-0,23 p<0,001). Esse achado é corroborado por outros estudos.^33^ Outros indicadores que refletem a lesão da microcirculação, como, por exemplo, a resolução da elevação do segmento ST e quantificação do *blush* miocárdico, poderiam melhorar o nosso modelo.^34^

De acordo com dados publicados na literatura, a taxa de reinfarto nos pacientes tratados com ATC é menor do que nos pacientes tratados por fibrinólise.^8 , 35^ Na nossa casuística a taxa foi de 0,5%. Esse achado é compatível com estudos randomizados, comparando a ATC com fibrinólise.^8^ Apesar da incidência de reinfarto ter sido relativamente baixa, a chance de óbito foi cerca de cinco vezes maior nos pacientes que tiveram este evento.

Em nosso estudo, encontramos correlação inversa entre o número de lesões tratadas e a chance de morte. Estudos prévios sugerem que a revascularização de outros vasos além daquele diretamente relacionado ao IAM não parece interferir de forma significativa nas chances de morte e reinfarto.^36^ Portanto, especulamos que o motivo mais provável, seria um viés de seleção, em que pacientes de menor risco teriam sido selecionados para o tratamento intervencionista adicional. Entretanto, não podemos excluir a hipótese de que a intervenção seletiva em obstruções de alto risco possa ter melhorado o prognóstico.

Outro achado inesperado foi o potencial efeito protetor da dislipidemia. No estudo TIMI, o uso de drogas hipolipemiantes também foi associado a melhor evolução.^10^ A explicação para esta descoberta conhecida como “paradoxo lipídico” não é completamente conhecida. Supõe-se que pacientes que referem dislipidemia, tem maior probabilidade de usar medicações, bem como ter mais cuidado com a própria saúde. Por outro lado, o achado de baixos níveis de lipoproteínas de baixa densidade (LDL) pode levar a uma menor prescrição de estatinas.^37 , 38^

Outros trabalhos, incluindo uma meta-análise de estudos randomizados^39^ e um modelo de risco,^25^ demonstraram o impacto do acesso radial na redução da mortalidade. Nosso modelo não corroborou esses achados. Uma possível explicação é a característica da nossa amostra. Nós excluímos pacientes tratados previamente com fibrinolíticos e tivemos uma baixa utilização de inibidores da glicoproteína IIb/IIa. Além disso, nossos operadores selecionaram o acesso baseado nas características clínicas do paciente e na sua própria expertise, levando a melhores resultados.

Dentre os modelos de risco apresentados na Tabela 3 , nosso trabalho foi um dos poucos a mencionar o sexo feminino como fator de risco ao óbito em pacientes tratados por ATC. Esse achado reforça a necessidade de um diagnóstico mais rápido e preciso e a adoção de estratégias de tratamento diferenciadas para as mulheres. Outro achado interessante, foi o pseudoefeito “protetor” da dislipidemia, como discutido, este achado sugere fortemente que os pacientes sem dislipidemia devem receber estatinas, nas mesmas doses preconizadas, independentemente do nível de colesterol, conforme indicado nas diretrizes.

Medidas com o objetivo de atenuar a lesão de reperfusão, podem diminuir ainda mais a taxa de mortalidade, pois, como demonstrado, além do fluxo TIMI III a função ventricular foi um importante marcador de bom prognóstico. Finalmente, o uso dos novos antiagregantes, associado a novos materiais e técnicas de intervenção podem reduzir a trombose do *stent* e consequentemente diminuir a letalidade.

### Limitações do estudo

Nosso estudo apresenta algumas limitações: trata-se de um estudo observacional, não randomizado, portanto avaliamos a associação entre o óbito e variáveis clínicas, angiográficas e complicações e não causalidade. Além disso, as variáveis foram coletadas de uma fonte secundária, fruto de contribuição espontânea, portanto, não foi possível a adjudicação dos dados. Finalmente o estudo apresenta falta de uniformidade em algumas definições e ausência de variáveis importantes relacionadas ao IAM, observa-se que o registro CENIC é rico em variáveis angiográficas e relativamente pobre em variáveis clínicas, justamente por ter sido concebido por intervencionistas.

Encontramos ainda uma baixa taxa de mortalidade hospitalar, o que sugere subnotificação, situação comumente encontrada em registros não mandatórios e não ligados a reembolso o que pode ter gerado viés de inclusão.

Outra limitação foi a presença de dados faltantes. Na tabela 1 variáveis com n diferente da amostra são assinaladas com asterisco. Observamos baixa perda de dados na maioria das variáveis. A variável função ventricular pela angiografia, apresentou elevado *missing* , porém a ventriculografia tem sido cada vez menos empregada na prática clínica e nosso estudo é reflexo do “mundo real”. Outra variável com perda significativa foi o diâmetro do vaso, que, talvez, tenha ocorrido por dificuldade de medida relacionada ao fato de o vaso estar ocluído na grande maioria dos casos.

## Conclusão

Os preditores de mortalidade nos pacientes submetidos a ATC primária catalogados no registro CENIC foram: classificação de Killip, reinfarto, idade avançada, disfunção global acentuada do VE, sexo feminino e fluxo TIMI 0/I pós-intervenção. Essa identificação dos elementos associados ao pior prognóstico, pode ser útil na estratificação e cuidados ao paciente coronariopata.

## * Material suplementar

Para informação adicional, por favor, clique aqui .
