# Supplementary material for: Preditores de Mortalidade Hospitalar nos Pacientes Tratados por Angioplastia Primária: Um Estudo de Caso-Controle Multicêntrico
Source: Arq Bras Cardiol. 2022 Jul 28;119(3):448–57. [Article in Portuguese] doi: 10.36660/abc.20210015 (PMC9438550; doi:10.36660/abc.20210015)
Supplement: Translation

# Translation: Predictors of Hospital Mortality Based on Primary Angioplasty Treatment: A Multicenter Case-Control Study

**Authors:** Pedro Paulo Neves de Castro, Marco Antonio Nazaré Castro, Guilherme Abreu Nascimento, Isabel Moura, José Luiz Barros Pena

**Affiliations:** 1 Brasil Hospital Marcio Cunha – Hemodinâmica, Ipatinga, MG – Brasil; 2 Brasil Faculdade de Ciências Médicas de Minas Gerais – Programa de Pós-Graduação stricto sensu em Ciências da Saúde, Belo Horizonte, MG – Brasil; 3 Brasil Unimed Vale do Aço, Ipatinga, MG – Brasil; 4 Brasil Hospital Felício Rocho – Ecocardiografia, Belo Horizonte, MG – Brasil

**Keywords:** Acute Myocardial Infarction, Database, Myocardial Reperfusion, Percutaneous Coronary Intervention, Mortality

## Abstract

**Background:**

Identification of high-risk patients undergoing primary angioplasty (PCI) is essential.

**Objective:**

Identify factors related to the causes of death in PCI patients.

**Methods:**

This work consisted of a multicenter case-control study using a Brazilian registry of cardiovascular interventions as the data source. The association between each variable and death was assessed using a binary logistic regression model, p <0.05 was considered significant.

**Results:**

A total of 26,990 records were analyzed, of which 18,834 (69.8%) were male patients, with a median age of 61 (±17) years. In the multivariate analysis, the main variables related to the causes of death with their respective odds ratios and 95%confidence intervals (CI) were advanced age, 70-79 years (2.46; 1.64-3.79) and ≥ 80 years (3.69; 2.38-5.81), p<0.001; the classification of Killip II (2.71; 1.92-3.83), Killip III (8.14; 5.67-11.64), and Killip IV (19.83; 14.85-26.69), p<0.001; accentuated global dysfunction (3.63; 2,39-5.68), p<0.001; and the occurrence of infarction after intervention (5.01; 2.57-9.46), p<0.001. The main protective factor was the post-intervention thrombolysis in myocardial infarction (TIMI) III flow (0.18; 0.13-0.24), p<0.001, followed by TIMI II (0.59; 0.41 -0.86), p=0.005, and male (0.79; 0.64-0.98), p = 0.032; dyslipidemia (0.69; 0.59-0.85), p<0.001; and number of lesions treated (0.86; 0.9-0.94), p<0.001.

**Conclusion:**

The predictors of mortality in patients undergoing PCI were Killip’s classification, reinfarction, advanced age, severe left ventricular dysfunction, female gender, and post-intervention TIMI 0 / I flow.

## Introduction

Cardiovascular disease (CVD) is the leading cause of morbidity and mortality in Brazil. Cardiac conditions account for 8.3% of all hospitalizations and 18.6% of all hospital expense reimbursements in the Brazilian public health system. Ischemic heart disease is the leading CVD cause of death.^1^

Access to treatment restoring coronary flow is essential in reducing mortality from ST-Elevation Myocardial Infarction (STEMI). Studies have shown a significant reduction in early mortality using aspirin with fibrinolytic medications.^2 - 4^

Another treatment method, primary percutaneous coronary intervention angioplasty (PCI), consists of the mechanical opening of the artery related to STEMI. It is the preferred treatment strategy if performed by an experienced team up to ninety minutes after admission.^5 - 7^ Compared to chemical fibrinolysis, PCI is considered the most effective treatment, and can reduce mortality rates, nonfatal infarction recurrence, and stroke.^8^

Identifying high-risk patients is essential for prognostic information and aids in the medical decision-making process. Knowing these variables can help select patients with a higher rate of events for future studies, adjust population baseline characteristics in epidemiological studies, and generate hypotheses for further studies.^9 , 10^

Several publications present models for risk stratification, but little data refer to the Brazilian population.^11 - 16^ In 1991, the National Cardiovascular Intervention Center (CENIC) was created, an official database of the Brazilian Society of Hemodynamics and Interventional Cardiology (SBHCI). This database contains information that comes from the spontaneous contribution of its members and has been used in other key publications in the literature.^17 - 19^

The present study aims to identify the risk factors for death in Brazilian patients undergoing PCI.

## Methods

This study used a secondary data source (CENIC) in a multicenter case-control study. Patients were divided into two groups: those who survived the procedure (controls) and those who died (for any reason). Data were collected during the hospitalization period.

### Population

Records of patients undergoing primary PCI were selected from January 2004 to December 2018. The exclusion criteria involved patients younger than 18 years of age or an unknown age, missing data on hospital mortality, and previous use or unknown use of thrombolytics.

This study also excluded patients submitted to procedures unapproved for primary angioplasty, according to the Brazilian Society of Cardiology Guidelines,^7^ including cases that used rotational, directional atherectomy; cutting balloon; and excimer laser devices. Altogether, 109 records reported at least one of these techniques.

From 29,003 original records, 26,990 were included in the analysis. The flowchart with the study population, exclusion criteria, and distribution of cases and controls is shown in Figure 1 .

**Figure 1:** Population, exclusion criteria, and distribution of cases and controls. BSC: Brazilian Society of Cardiology; PCI: primary angioplasty.

## Definitions

Patients with clinical and electrocardiographic criteria compatible with the diagnosis of STEMI, selected for a primary angioplasty strategy, were included. The diagnosis was confirmed by angiography in all cases. The decision to include patients in the registry was at the discretion of the interventional cardiologist.

Analysis regarding the angiographic variables, including ventricular function, was visually estimated by the examiners. The definitions followed the SBHCI Guidelines for Percutaneous Coronary Intervention and Adjunct Diagnostic Methods in Interventional Cardiology.^20^

The choice of vascular access, use of adjuvant medications, and procedure techniques were chosen by the examiners.

Coreware managed the CENIC registry, performed the research data extraction, and maintained participants and hospitals of origin confidential (www.coreware.com.br).

The variables were selected based on previous publications.^10 - 16^

### Statistical analysis

Qualitative variables were presented as frequencies and quantitative variables as medians (interquartile range). Quantitative variables were subjected to the Kolmogorov-Smirnov normality test. The comparison of mortality rates between genders was evaluated using the chi-square test. The association between each predictor variable and death outcome was assessed using a simple logistic regression model. The univariate analysis was performed with all variables shown in Table 1 . These variables were selected based on previous studies. Variables with p<0.20 in the univariate analysis were included in a multivariate binary logistic regression model. The final model was obtained using the stepwise strategy, and the quality of the adjustment was assessed using the Hosmer-Lemeshow test. The missing data were not considered in the statistical analysis.

**Table 1:** Sample characteristics and association with the outcome of death

Features	All sample (n = 26,990)	No (n = 26,086)	Yes (n = 904)	p-value	OR (95% CI)
**Gender**					
F	8.156 (30.2%)	7.764 (29.8%)	392 (43.4%)	-	-
M	18.834 (69.8%)	18.322 (70.2%)	512 (56.6%)	<0.001	0.55 (0.48; 0.63)
**Age**					
19 to 49 years	4.472 (16.6%)	4.400 (16.9%)	72 (8.0%)	-	-
50 to 59 years	7.886 (29.2%)	7.734 (29.6%)	152 (16.8%)	0.204	1.20 (0.91; 1.60)
60 to 69 years	7.395 (27.4%)	7.166 (27.5%)	229 (25.3%)	<0.001	1.95 (1.50; 2.57)
70 to 79 years	4.968 (18.4%)	4.714 (18.1%)	254 (28.1%)	<0.001	3.29 (2.54; 4.32)
≥ 80 years	2.269 (8.4%)	2.072 (7.9%)	197 (21.8%)	<0.001	5.81 (4.44; 7.69)
Killip* (n = 26,989)					
I	20.560 (76.2%)	20.359 (78.0%)	201 (22.2%)	-	-
II	3.560 (13.2%)	3.452 (13.2%)	108 (11.9%)	<0.001	3.17 (2.49; 4.01)
III	1.079 (4.0%)	969 (3.7%)	110 (12.2%)	<0.001	11.50 (9.01; 14.60)
IV	1.790 (6.6%)	1.305 (5.0%)	485 (53.7%)	<0.001	37.64 (31.69; 44.86)
Lesion location* (n = 27,179)					
Proximal LAD	7.266 (26.9%)	6.951 (26.6%)	315 (34.8%)	-	-
Middle / distal right coronary and branches	6.451 (23.9%)	6.326 (24.3%)	125 (13.8%)	<0.001	0.44 (0.35; 0.54)
Middle / distal LAD and branches	5.515 (20.4%)	5.379 (20.6%)	136 (15.0%)	<0.001	0.56 (0.45; 0.68)
Proximal right coronary	3.696 (13.7%)	3.561 (13.7%)	135 (14.9%)	0.089	0.84 (0.68; 1.03)
Distal circumflex / branches	1.989 (7.4%)	1.949 (7.5%)	40 (4.4%)	<0.001	0.45 (0.32; 0.62)
Proximal circumflex	1.486 (5.5%)	1.423 (5.5%)	63 (7.0%)	0.869	0.98 (0.73; 1.28)
Grafts	370 (1.4%)	345 (1.3%)	25 (2.8%)	0.029	1.60 (1.02; 2.39)
Left main	217 (0.8%)	152 (0.6%)	65 (7.2%)	<0.001	9.44 (6.87; 12.83)
Disease extent* (n = 26,751)					
Single arterial	12.699 (47.5%)	12.484 (48.3%)	215 (24.0%)	-	-
Biarterial	7.889 (29.5%)	7.610 (29.4%)	279 (31.1%)	<0.001	2.13 (1.78; 2.55)
Multiarterial + LMCA	36 (0.1%)	29 (0.1%)	7 (0.8%)	<0.001	14.02 (5.60; 30.59)
Left main	44 (0.2%)	29 (0.1%)	15 (1.7%)	<0.001	30.03 (15.48; 55.99)
Triarterial	6.083 (22.7%)	5.702 (22.1%)	381 (42.5%)	<0.001	3.88 (3.28; 4.61)
Door-to-balloon time^1^* (minutes) (n = 25,837)	70.00 (75.00)	70.00 (75.00)	80.00 (66.80)	0.010	1.0006 (1.0001; 1.001)
Previous CABG surgery	803 (3.0%)	759 (2.9%)	44 (4.9%)	<0.001	1.71 (1.23; 2.30)
Previous angioplasty	3.143 (11.6%)	3.044 (11.7%)	99 (11.0%)	0.508	0.93 (0.75; 1.14)
Previous AMI* (n = 26,957)	2.948 (10.9%)	2.808 (10.8%)	140 (15.5%)	<0.001	1.52 (1.26; 1.82)
Diabetes Mellitus	5.270 (19.5%)	5.021 (19.2%)	249 (27.5%)	<0.001	1.59 (1.37; 1.85)
Insulin-dependent	753 (2.8%)	697 (2.7%)	56 (6.2%)	<0.001	2.41 (1.80; 3.16)
Hypertension	19.045 (70.6%)	18.406 (70.6%)	639 (70.7%)	0.934	1.006 (0.87; 1.17)
ARF	43 (0.2%)	25 (0.1%)	18 (2.0%)	<0.001	21.18 (11.35; 38.75)
Smoking	9.521 (35.3%)	9.273 (35.5%)	248 (27.4%)	<0.001	0.69 (0.59; 0.79)
Dyslipidemia	13.221 (49.0%)	12.825 (49.2%)	396 (43.8%)	0.002	0.81 (0.70; 0.92)
Family history	6.364 (23.6%)	6.208 (23.8%)	156 (17.3%)	<0.001	0.67 (0.56; 0.79)
**TIMI Pre**					
0	18.160 (67.3%)	17.472 (67.0%)	688 (76.1%)	-	-
1	1.576 (5.8%)	1.513 (5.8%)	63 (7.0%)	0.678	1.06 (0.81; 1.36)
2	2.435 (9.0%)	2.371 (9.1%)	64 (7.1%)	0.004	0.69 (0.52; 0.88)
3	4.819 (17.9%)	4.730 (18.1%)	89 (9.8%)	<0.001	0.48 (0.38; 0.59)
TIMI Post* (n = 26,975)					
0	1.175 (4.4%)	955 (3.7%)	220 (24.4%)	-	-
1	322 (1.2%)	257 (1.0%)	65 (7.2%)	0.554	1.10 (0.80; 1.49)
2	1.289 (4.8%)	1.146 (4.4%)	143 (15.9%)	<0.001	0.54 (0.43; 0.68)
3	24.189 (89.7%)	23.715 (91.0%)	474 (52.5%)	<0.001	0.09 (0.07; 0.10)
Diameter of vessel^1^* (n = 19,931)	3.00 (0.75)	3.00 (0.75)	3.00 (0.75)	<0.001	0.63 (0.52; 0.76)
LV function* (n = 16,880)					
Normal	3.169 (18.8%)	3.139 (19.2%)	30 (6.0%)	-	-
Mild global dysfunction	6.167 (36.5%)	6.123 (37.4%)	44 (8.8%)	0.230	0.75 (0.47; 1.21)
Moderate global dysfunction	5.230 (31.0%)	5.130 (31.3%)	100 (20.0%)	<0.001	2.04 (1.37; 3.13)
Marked global dysfunction	2.314 (13.7%)	1.989 (12.1%)	325 (65.1%)	<0.001	17.10 (11.92; 25.47)
Minor vascular complications	87 (0.3%)	82 (0.3%)	5 (0.6%)	0.219	1.76 (0.62; 3.94)
Major vascular complications	31 (0.1%)	25 (0.1%)	6 (0.7%)	<0.001	6.97 (2.58; 15.94)
Hemorrhagic stroke	16 (0.1%)	12 (<0.1%)	4 (0.4%)	<0.001	9.66 (2.70; 27.78)
Ischemic stroke	17 (0.1%)	11 (<0.1%)	6 (0.7%)	<0.001	15.84 (5.45; 41.72)
Access site* (n = 25,032)					
Femoral	19.278 (77.0%)	18.690 (76.7%)	588 (86.6%)	-	
Brachial - dissection	299 (1.2%)	291 (1.2%)	8 (1.2%)	0.709	0.87 (0.39; 1.66)
Brachial - puncture	165 (0.7%)	162 (0.7%)	3 (0.4%)	0.364	0.59 (0.15; 1.55)
Radial	5.290 (21.1%)	5.210 (21.4%)	80 (11.8%)	<0.001	0.49 (0.38; 0.61)
Abxicimab* (n = 25,107)	830 (3.3%)	800 (3.3%)	30 (4.4%)	0.103	1.36 (0.92; 1.94)
Tirofiban* (n = 25,107)	3.199 (12.7%)	3.067 (12.6%)	132 (19.4%)	<0.001	1.68 (1.38; 2.03)
AAS* (n = 25,107)	22.475 (89.5%)	21.873 (89.5%)	602 (88.5%)	0.394	0.90 (0.71; 1.15)
Calcification	5.448 (20.2%)	5.176 (19.8%)	272 (30.1%)	<0.001	1.74 (1.50; 2.01)
Intracoronary thrombus	16.812 (62.3%)	16.197 (62.1%)	615 (68.0%)	<0.001	1.30 (1.13; 1.50)
Reinfarction	130 (0.5%)	98 (0.4%)	32 (3.5%)	<0.001	9.73 (6.40; 14.42)
Obstructions treated^1^	1.00 (1.00)	1.00 (1.00)	1.00 (0.00)	<0.001	0.84 (0.79; 0.89)

*variables that presented missings, n valid is in parentheses.^1^Data presented as median (interquartile range). The p-values refer to the simple binary logistic model. LAD: left anterior descending artery; LMCA: left main coronary artery; CABG: coronary artery bypass graft; SAH: systemic arterial hypertension; ARF: acute renal failure; LV: left ventricle; ASA: acetylsalicylic acid. Source: The author, 2021.

Results were presented as odds ratios (OR) with the respective 95% confidence intervals (95% CI). The analyses were performed using the free R program, version 4.0.0, and p <0.05 was considered significant.

### Ethical aspects

The research was approved by the Research Ethics Committee of Faculdade de Ciências Médicas de Minas Gerais, logged under protocol number: 3.502.883. The need for free and informed consent forms was waived. All procedures in this study were in accordance with resolution 466/2012.

## Results

A total of 26,990 records were analyzed, from all Brazilian regions; the distribution of cases is shown in Figure 2 . Most of the records, 1,883 (69.8%) were male, with a median age of 61 (±17) years, and the most frequent risk factor was systemic arterial hypertension, reported by 19,045 (70.6%) participants.

**Figure 2:** Distribution of cases by region.

Most of the patients, 20,560 (76.2%), presented a Killip class I classification, while 12,699 (47.5%) presented a predominance of disease affecting a single vessel and 6,167 (36.5%) presented mild ventricular dysfunction.

The total number of deaths was 904 patients, and the overall mortality rate was 3.3%. The mortality rate was lower in males than in females (2.7% and 4.8%, respectively, p<0.001).

Table 1 shows all sample characteristics, their association with death outcomes, and the results of the OR association test with a 95%CI, with respective p-values obtained by adjusting univariate logistic models. In this univariate analysis, the variables with p<0.20 used in the multiple models were gender, age, Killip classification, location of lesions, extent of coronary disease, door-to-balloon time, personal history of coronary bypass surgery, report of infarction, diabetes, smoking, dyslipidemia, systemic arterial hypertension, family history of early coronary disease, classification of TIMI flow before and after the intervention, vessel diameter, the development of major vascular complications, renal failure and ischemic hemorrhagic stroke, reinfarction, vascular access, the average number of obstructions treated and the presence of calcification, and thrombus.

Table 2 shows the factors related to the death outcomes, OR association test with a 95% CI, and p-values obtained by adjusting the multivariate binary logistic regression model.

**Table 2:** Variables that correlated significantly and independently with in-hospital death

Feature	OR	CI 95% OR	p-value
Intercept	0.021	(0.011; 0.039)	<0.001
Gender M	0.789	(0.635; 0.981)	0.032
**Age (ref. <50)**			
50 to 59	1.625	(1.059; 2.540)	0.029
60 to 69	2.004	(1.336; 3.076)	0.001
70 to 79	2.462	(1.635; 3.789)	<0.001
≥ 80	3.688	(2.384; 5.812)	<0.001
**Killip (ref. I)**			
II	2.718	(1.919; 3.827)	<0.001
III	8.139	(5.672; 11.637)	<0.001
IV	19.833	(14.851; 26.688)	<0.001
Dyslipidemia	0.689	(0.558; 0.850)	<0.001
**TIMI Post (ref. 0)**			
1	1.303	(0.774; 2.162)	0.313
2	0.593	(0.409; 0.857)	0.005
3	0.176	(0.133; 0.235)	<0.001
**LV function (ref. Normal)**			
Mild global dysfunction	0.799	(0.491; 1.322)	0.373
Moderate global dysfunction	1.206	(0.782; 1.914)	0.410
Marked global dysfunction	3.625	(2.393; 5.675)	<0.001
Infarction after intervention	5.006	(2.568; 9.460)	<0.001
Number of lesions treated	0.859	(0.785; 0.938)	<0.001

*Hosmer-Lemeshow, p-value 0.683. LV: left ventricle. Source: The author, 2021.*

## Discussion

The main mortality indicators in patients submitted to primary PCI found in the present study, in addition to age and female gender, were related to the impact of infarction on ventricular function, such as the Killip classification and the presence of marked LV global dysfunction analyzed by angiography. On the other hand, the presence of TIMI II/III flow after the intervention reflected the success of the treatment, which seeks precisely to maintain ventricular function and prevent other cardiovascular complications. The occurrence of reinfarction was rare, but it proved to be an independent indicator of mortality in these patients.

Mortality rates in patients undergoing PCI vary from 2.3% to 11.9%, according to different sources.^15 , 21 - 24^ The present study’s database identified a 3.4% death rate. This finding may be related to underreporting and the lower risk of the sample. Table 3 shows the comparison between variables correlated to the death outcome in our study with others published in the literature.^11 - 14 , 25 , 26^

**Table 3:** Comparison of variables related to death outcomes

	CENIC (n=26,990)	DynTIMI (n=20,506)	PAMI (n=3,252)	CADILLAC (n=2,082)	GRACE (n=11,389)	Zwolle (n=1,791)	ALPHA (n=1,255)
**Time**	**Hospital**	**One year**	**Six months**	**One year**	**Six months**	**30 days**	**30 days**
Age	+	+	+	+	+	+	+
Female	+						
Arterial Hypotension		+			+		+
Heart Rate		+	+		+		+
Killip classification	+	+	+	+	+		
Diabetes mellitus			+				
Hypertension							
Angina pectoris							
Previous AMI or BBB		+	+			+	
Weight		+				+	
Ischemia time		+					
Flow (final TIMI from 0 to 2)	+			+		+	
LVEF				+			
Marked LV Dysfunction	+						
Anemia				+			
Three vessel disease				+		+	
ST-segment deviation					+		
Creatinine / ARF		+		+	+		
Cardiac arrest					+		+
Myocardial injury markers					+		
Infarction recurrence	+	+					
Stroke		+					
Arrhythmia		+					
HF / Shock		+				+	
Major bleeding		+					
Femoral access							+

*CENIC: National Cardiovascular Intervention Center; dynTIMI: dynamic Thrombolysis In Myocardial Infarction; PAMI: Primary Angioplasty in Myocardial Infarction; CADILLAC: Controlled Abciximab and Device Investigation to Lower Late Angioplasty Complications; GRACE: Global Registry of Acute Coronary Events; ALPHA: (Age, Life support, Pressure, Heart rate, Access site); SAH: systemic arterial hypertension; AMI: acute myocardial infarction; LBBB: left bundle branch block; LVEF: left ventricular ejection fraction; LV: left ventricle; SBP: systolic blood pressure; ARF: acute renal failure; HF: heart failure. Source: The author, 2021.*

The present study found that the only indicator of the CENIC study that differs from the other risk models presented in Table 3 was the female sex. However, this finding has already been reported by other publications.^27 , 28^

Some authors report the more significant presence of atypical symptoms in females who delay their treatment, the so-called Yentl syndrome. Angioplasty can also be more challenging, leading to a lower success rate.^29^ Total ischemia time, other bleeding complications outside the access site, and weight were missing from our database, which could partly explain this worse outcome in women.

The Killip and Kimball classification was the variable that proved to be the best prognostic indicator, a fact corroborated by other studies.^10 , 12 , 13^ In the Grace registry, the chance of death increased nearly three-fold with each increase in the Killip classification, 3.30 (3.00-3.60), p <0.001. The present study’s series showed 1,790 cases (6.6% of the total) with Killip class IV (cardiogenic shock), similar to the incidence described in the literature (5 to 10%).^30^

Ventricular failure is the leading cause of death in these patients, and the only effective treatment is early reperfusion. The use of ventricular assist devices, such as the intra-aortic balloon, has conflicting results.^31^ Other devices have been tested and even used in clinical practice, but no conclusive studies have been published in the literature.^32^

The purpose of the intervention is to obtain the final TIMI III flow. This result was strongly related to reducing the chances of death (OR 0.18; CI 0.13-0.23, p <0.001). Other studies also corroborate this finding.^33^ Other indicators that reflect the microcirculation injury, such as the resolution of the elevation of the ST segment and the quantification of the myocardial blush, was able to improve our model.^34^

According to published data in the literature, the reinfarction rate in patients treated with primary angioplasty is lower than in those receiving fibrinolysis as a reperfusion strategy.^35^ In our sample, the rate was 0.5%. This finding is compatible with randomized studies, comparing PCI with fibrinolysis.^8^ Although reinfarction incidence was relatively low, the chance of death was about five-fold higher in patients who experienced this event.

The present study identified an inverse correlation between the number of lesions treated and the chance of death. Previous studies suggest that the revascularization of vessels other than those directly related to AMI does not seem to significantly interfere with the chances of death and reinfarction.^36^ We speculate that the most likely reason would be a selection bias, where lower-risk patients would have eventually been selected for additional interventional treatment. However, the hypothesis that selective intervention in high-risk obstructions may have improved the results is impossible to rule out.

Another unexpected finding was the potential protective effect of dyslipidemia. In the TIMI study, the use of lipid-lowering drugs was also associated with a better evolution.^10^ The explanation for this discovery, known as the “lipid paradox,” is not entirely known. It is assumed that patients who report dyslipidemia are more likely to take medications and care for their health. On the other hand, the finding of low levels of low-density lipoprotein (LDL) may lead to a lower prescription of statins.^37 , 38^

Several trials, including a meta-analysis of randomized studies^39^ and a risk model,^25^ have demonstrated the impact of radial access in reducing mortality. Our model did not corroborate these findings, which can possibly be explained by the study’s sample characteristics. Cases with a previous use of fibrinolytic medications were excluded, and a low rate of glycoprotein IIb / IIIa inhibitors was found. Moreover, our study’s operators likely selected the access site based on patients’ clinical characteristics and operator procedural expertise, thus leading to better results.

Among the risk models presented in Table 3 , ours was the only one that showed an association with the female gender as a risk factor for mortality in patients treated for PCI. This finding reinforces the need for a faster, more accurate diagnosis and adoption of different treatment strategies in females. Another interesting result was the pseudo “protective” effect of dyslipidemia. As discussed, this finding strongly suggests that patients without dyslipidemia should receive statins in the same recommended doses, regardless of the cholesterol levels indicated in the guidelines.

Measures to attenuate reperfusion injury can further decrease the mortality rate, since, as demonstrated, in addition to the TIMI III flow, ventricular function was an important marker of good prognosis. Finally, new antiplatelet agents, combined with new intervention materials and techniques, can reduce stent thrombosis and decrease mortality.

### Study limitations

The present study does have some limitations. It is an observational, non-randomized study, which assessed the association between death and clinical, angiographic variables, complications, and non-causality. Additionally, the variables were collected from a secondary source, resulting from spontaneous contributions; therefore, it was impossible to properly judge the data. Finally, the study lacked uniformity in definitions of some variables related to AMI. It was observed that the CENIC record was rich in angiographic variables and relatively poor in clinical variables, precisely because it was conceived by interventionists.

A low rate of hospital mortality was also observed, which suggests underreporting, a situation commonly found in nonmandatory records and not linked to reimbursement, which may have generated inclusion bias.

Another limitation was the presence of missing data. In Table 1 , the variables with n different from the sample are marked with an asterisk. Low data loss was observed in most variables. The variable of ventricular function by angiography presented a high level of *missing* . However, ventriculography has been less and less used in clinical practice, and the present study better reflects the “real world”. Another variable with significant loss was the diameter of the vessel, which may have occurred due to measurement difficulty related to the fact that the vessel was occluded in most cases.

## Conclusion

The predictors of mortality in patients undergoing primary PCI cataloged in the CENIC registry were: Killip classification, reinfarction, advanced age, severe systolic dysfunction of the left ventricle, female gender, and postintervention TIMI 0 / I flow. This identification of the worst prognosis elements can be useful in stratifying and caring for coronary patients.

## * Supplemental Materials

For additional information, please click here .
